# Local Regeneration of Dentin-Pulp Complex Using Controlled Release of FGF-2 and Naturally Derived Sponge-Like Scaffolds

**DOI:** 10.1155/2012/190561

**Published:** 2011-11-17

**Authors:** Chiaki Kitamura, Tatsuji Nishihara, Masamichi Terashita, Yasuhiko Tabata, Ayako Washio

**Affiliations:** ^1^Division of Pulp Biology, Operative Dentistry and Endodontics, Department of Cariology and Periodontology, Kyushu Dental College, Manazuru 2-6-1, Kokurakita, Kitakyushu 803-8580, Japan; ^2^Division of Infections and Molecular Biology, Department of Health Promotion, Kyushu Dental College, Manazuru 2-6-1, Kokurakita, Kitakyushu 803-8580, Japan; ^3^Division of Comprehensive Dentistry, Department of Clinical Communication and Practice, Kyushu Dental College, Manazuru 2-6-1, Kokurakita, Kitakyushu 803-8580, Japan; ^4^Department of Biomaterials, Institute for Frontier Medical Sciences, Kyoto University, 53 Kawara-cho, Shogoin, Sakyo-ku, Kyoto 606-8507, Japan

## Abstract

Restorative and endodontic procedures have been recently developed in an attempt to preserve the vitality of dental pulp after exposure to external stimuli, such as caries infection or traumatic injury. When damage to dental pulp is reversible, pulp wound healing can proceed, whereas irreversible damage induces pathological changes in dental pulp, eventually requiring its removal. Nonvital teeth lose their defensive abilities and become severely damaged, resulting in extraction. Development of regeneration therapy for the dentin-pulp complex is important to overcome limitations with presently available therapies. Three strategies to regenerate the dentin-pulp complex have been proposed; regeneration of the entire tooth, local regeneration of the dentin-pulp complex from amputated dental pulp, and regeneration of dental pulp from apical dental pulp or periapical tissues. In this paper, we focus on the local regeneration of the dentin-pulp complex by application of exogenous growth factors and scaffolds to amputated dental pulp.

## 1. Limitations of Conventional Therapy for Preservation of Dental Pulp

Dental pulp is sometimes affected by external stimuli such as caries infection or traumatic injury. Preservation of dental pulp and maintenance of its viability are essential to avoid tooth loss, and dentists carry out restorative procedures with pulp capping to regulate inflammatory responses of dental pulp, or cement lining on a cavity floor to block external stimuli. Reversible damage induces pulp wound healing, and direct pulp capping and pulpotomy with calcium hydroxide are known to be effective to induce pulp wound healing mechanisms.

 After external stimuli such as cavity preparation, apoptosis of pulp cells including odontoblasts is induced [1–5], followed by pulp wound healing including reactionary and reparative dentinogenesis. Reactionary dentin is formed by surviving odontoblasts, whereas reparative dentin is formed by odontoblast-like cells that are differentiated from pulp cells of residual dental pulp, resulting in a reduction in dental pulp size and vitality [6–8].

 When the external damage to dental pulp induces irreversible changes of the pulp, dentists carry out pulpectomy. Generally, a root canal after pulpectomy is tightly filled with biomaterials such as gutta-percha to prevent reinfection by bacteria. However, a tooth without vital dental pulp has lost its defensive ability, which is often followed by the severe damage such as the progression of deep radicular caries or tooth facture, resulting in extraction of the tooth. Furthermore, a treated tooth is often reinfected by bacteria because of its complicated anatomical structure or inadequate treatment by a dentist, resulting in formation of a lesion around the root apex with bone resorption. The success rate of the endodontic retreatment is lower than that of pulpectomy [9–12]. To overcome these limitations of the present endodontic treatment, the preservation of dentin-pulp complex is the clear strategy. However, when a dentin defect and the resultant exposure of dental pulp tissue reach a critical size, no treatments available are able to preserve and maintain the vitality of dental pulp. It is considered important to develop regeneration therapy for dental pulp or the dentin-pulp complex.

## 2. Regeneration of the Dentin-Pulp Complex

**Figure 1:**
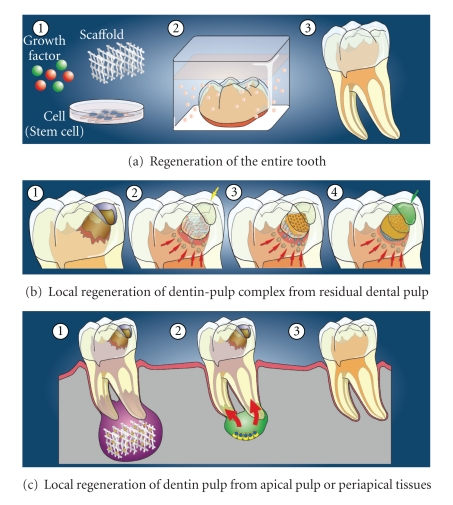
Strategies for regeneration of the dentin-pulp complex with three factors for tissue regeneration; growth factors, scaffolds, and cells (stem cells or progenitor cells). (a) Regeneration of the entire tooth. (b) Local regeneration of the dentin-pulp complex in the dentin defect area from residual dental pulp. (c) Local regeneration of dental pulp from apical dental pulp or periapical tissues.

It is well known that growth factors, such as bone morphogenetic proteins (BMPs) and fibroblast growth factors (FGFs), stem cells, and scaffolds, are essential for tissue engineering to regenerate tissues [13]. During regeneration processes, stem cells differentiate into specific cells for tissue defects, growth factors such as BMPs and FGFs induce proliferation and differentiation of stem cells, and scaffolds with properties of extracellular matrix temporally support structures for cell growth, differentiation, and tissue formation. In studies to develop the regeneration therapy of the dentin-pulp complex, three strategies that utilize these essential three factors have been proposed; regeneration of the entire tooth, local regeneration of the dentin-pulp complex in dentin defect area from residual dental pulp, and regeneration of dental pulp from apical dental pulp or periapical tissues including the periodontal ligament and bone (Figure 1).

### 2.1. Regeneration of Entire Tooth

Regeneration of the entire tooth is accepted as a model of organ replacement and regeneration therapy. Recently, it was reported that tooth germs can be bioengineered using a three-dimensional organ-germ culture method, in which dental epithelial and mesenchymal cells isolated from tooth germs were cultured in three-dimensional scaffolds for the replacement therapy. Scaffolds consisted of synthetic polymers such as poly (lactide-co-glycolide) (PLGA) and bioceramics such as hydroxyapatite, tricalcium phosphate and calcium carbonate hydroxyapatite were examined in the three-dimensional organ-germ culture [14–21]. It was also reported that bioengineered teeth were generated from three-dimensionally arranged dental epithelial and mesenchymal cells in collagen gels by *in vitro* cell aggregate and manipulation method, and that the bioengineered tooth germ generated a structurally correct tooth showing penetration of blood vessels and nerve fibers *in vivo* transplantation into mouse maxilla, resulting in a successful fully functioning tooth replacement [22–25]. These bioengineered teeth, however, were reconstructed with dental epithelial and mesenchymal cells from genuine tooth germs. Further research will be needed to regenerate the entire tooth from other cell sources such as induced pluripotent stem (iPS) cells.

### 2.2. Local Regeneration from Residual Dental Pulp

Local regeneration of the dentin-pulp complex from residual dental pulp has been mainly delivered by researchers who are engaged in clinical practice. Several studies have reported the use of local applications of bioactive molecules such as BMPs and recombinant fusion ameloblastin to exposed pulp [26–28]. However, local application of bioactive molecules without scaffolds only induces reparative dentin formation toward residual dental pulp, which is the same result provided by conventional therapy such as pulp capping. 

 Induction of appropriate pulp wound healing and formation of new dentin in dentin defects are essential for the local regeneration of the dentin-pulp complex and vital pulp therapies to form new dentin in defects. Several papers demonstrated the local regeneration of dentin-pulp complex in different methods. It was reported that BMP-4 with dentin powder induced dentinogenesis in dentin cavity with pulp exposure [29]. In this research, stem or progenitor cells were induced from residual pulp through the exposure site at the bottom of the cavity. It was also reported that ultrasound-mediated gene delivery of growth factors such as growth/differentiation factor 11 (GDF-11)/BMP-11 into dental pulp stem cells by *in vivo* sonoporation induced reparative dentinogenesis [30–32], and that the *ex vivo* gene therapy by the transplantation of pulp stem/progenitor cells transfected with some growth factors such as GDF-11/BMP-11 stimulated reparative dentinogenesis [33–36].

**Figure 2:**
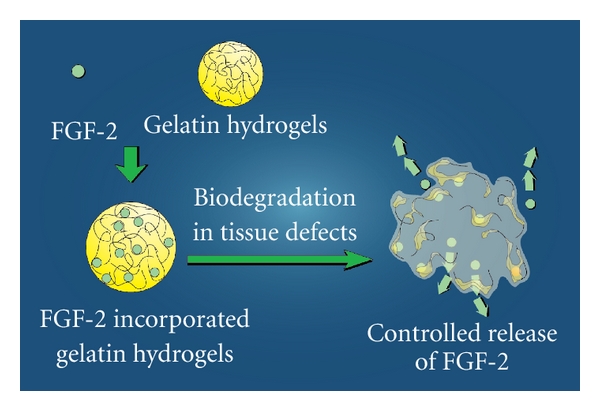
Controlled release of FGF-2. Gelatin hydrogels has an ability to incorporate growth factors such as FGF-2. After implantation of gelatin hydrogels incorporating FGF-2 with scaffolds, such as collagen sponge, FGF-2 is gradually released from gelatin hydrogels biodegraded by proteinase at tissue defect area. The controlled released FGF-2 can induce tissue regeneration.

**Figure 3:**
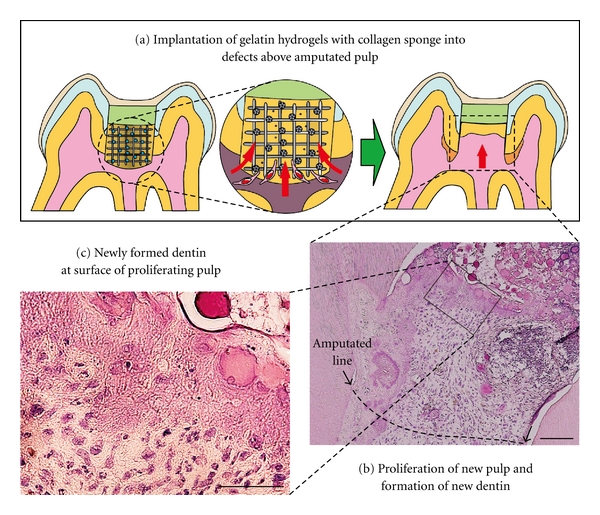
Local regeneration of the dentin-pulp complex in dentin defect area by implantation of gelatin hydrogels incorporating FGF-2. (a) Gelatin hydrogels incorporating FGF-2 with collagen sponge are implanted into dentin defect area. Controlled release of FGF-2 from biodegraded gelatin hydrogels can induce pulp stem cells or progenitor cells, as well as vessels, into collagen sponge at defect, resulting in the regeneration of pulp in the defect area and the formation of regenerative dentin on surface of the new pulp. (b) Histological photograph of proliferating pulp and newly regenerated dentin at surface of proliferating pulp. (c) High magnification of the regenerated dentin.

 FGF-2 is known to play a role in both physiological and pathological conditions [37–39]. It was previously demonstrated that a gradual and continual release of biologically active FGF-2 was achieved by *in vivo* biodegradation of gelatin hydrogels that incorporated FGF-2 [40–43] (Figure 2). Recently, we used FGF-2, gelatin hydrogels, and collagen sponge as a scaffold to induce local regeneration of rat dentin-pulp complex. We implanted free FGF-2 or gelatin hydrogels incorporating FGF-2 with collagen sponge into dentin defects above the amputated pulp of rat molars, and we found that a noncontrolled release of free FGF-2 only accelerated reparative dentin formation in the residual dental pulp, whereas a controlled release of FGF-2 from gelatin hydrogels induced formation of DMP-1-positive and nestin-negative osteodentin in the pulp proliferating in the dentin defects. Furthermore, the controlled release of an appropriate dosage of FGF-2 from gelatin hydrogels induced the formation of the dentinal bridge-like osteodentin on the surface of the regenerated pulp (Figure 3). These results suggest that our method inducing the regeneration of dentin and pulp into defect area from the amputated pulp is different from the conventional therapy that induces reparative dentinogenesis toward the amputated pulp [44, 45]. 

### 2.3. Local Regeneration from Periapical Tissues

Studies on regeneration of dental pulp from the apical area began from the identification of stem cells in the apical areas of developing teeth in which root formation is incomplete. It is suggested the existence of a new population of mesenchymal stem cells residing in the apical papilla (SCAPs) of incompletely developed teeth, and these cells have the ability to differentiate into odontoblast-like cells [46–48]. SCAPs play important roles in continued root formation, and they have been suggested to participate in pulp wound healing and regeneration. It is also known that bone-marrow-derived mesenchymal stem cells (BMMSCs) have multipotent abilities to differentiate into several cell types and undergo osteogenic differentiation. Periapical tissues include periodontal ligament, and bone marrow, which is the source of BMMSCs [49–54]. Localization of SCAPs and BMMSCs in the apical area indicate the possibility of the induction of these stem cells for the regeneration of the dentin-pulp complex.

## 3. Scaffolds for Regeneration of Dentin-Pulp Complex

It is important to select appropriate scaffolds for successful tissue regeneration. It is well known that essential properties of scaffolds are mechanical properties such as porous three-dimension structure, and mechanical strength, as well as biological properties such as biocompatibility and biodegradation [55]. In recent research and clinical approach, the following biomaterials are utilized for tissue regeneration therapy; polyethylene terephthalate, poly(L-lactide-*co*-D, L lactide), polylactic acid, polyglycolic acid, PLGA, polyvinyl alcohol, collagen, hyaluronic acid, hydroxyapatite, tricalcium phosphate, silk fibroin, bioactive glasses, and ceramic materials [56]. Of the variety of biomaterials tested, collagen sponge has been found to be well suited for the regeneration of bone defects, as collagen is a major component of the extracellular matrix. Also in the research field of tooth regeneration therapy, several lines of studies analyzed and discussed which three-dimensional scaffolds were suitable for the regeneration of dentin-pulp complex [57–60].

 Recently, we have been focusing on the application of hyaluronic acid for local regeneration of the dentin-pulp complex. Hyaluronic acid is one of the glycosaminoglycans present in the extracellular matrix and plays important roles in maintaining morphologic organization by preserving extracellular spaces, and it has been reported to have excellent potential for tissue engineering [61–65]. The roles of hyaluronic acid in some biological processes, including inhibition of inflammation and pain, and differentiation of osteoblastic and osteoclastic cells, were recently studied [66–68]. In addition, some researchers have reported that intra-articular hyaluronic acid treatment for patients with osteoarthritic knees reduced painful symptoms and improved joint mobility [69, 70].

 Dental pulp is a type of connective tissue derived from the dental papilla, and contains large amounts of glycosaminoglycans [71, 72]. Previously, the contribution of hyaluronic acid to the initial development of dentin matrix and dental pulp [73], *in vivo* application of hyaluronic acid gels on the wound healing processes of dental pulp, and the application of gelatin-chondroitin-hyaluronan tri-copolymer scaffold to dental bud cells were reported [74, 75].

**Figure 4:**
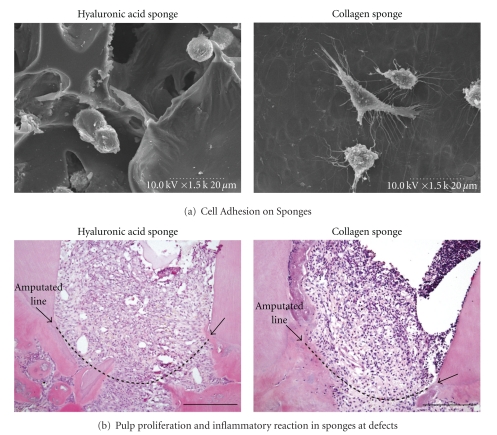
Application of hyaluronic acid sponge for local regeneration of the dentin-pulp complex. (a) KN-3 cells, odontoblastic progenitor cells, adhered to hyaluronic acid sponge, as well as collagen sponge. (b) Histological changes of amputated dental pulp after implantation of hyaluronic acid sponge *in vivo*. Amputated dental pulp well proliferated into hyaluronic acid and collagen sponges. Compared with collagen sponge, hyaluronic acid sponge significantly suppressed inflammatory reaction of dental pulp.

 To clarify whether hyaluronic acid sponge (molecular weight 800 kDa) is useful as a scaffold for wound healing and regeneration of dental pulp, we compared *in vitro* and *in vivo* effects of hyaluronic acid sponge and collagen sponge on KN-3 odontoblast-like cell line and amputated dental pulp of rat molars. KN-3 cells, which were established from dental pulp of rat incisors, have odontoblastic properties such as high alkaline phosphatase activity and calcification ability [76]. We found that KN-3 cells adhered to both hyaluronic acid and collagen sponges during the culture period. *In vivo* results following implantation of both sponges in dentin defect areas above the amputated pulp showed that dental pulp proliferation and invasion of vessels into the hyaluronic acid and collagen sponges were well induced from the amputated dental pulp. These results suggest that hyaluronic acid sponge has an ability to induce and sustain dental pulp tissue regenerated from residual amputated dental pulp. In addition, we found that the inflammatory responses of KN-3 cells and the amputated dental pulp to hyaluronic acid sponge were lower than those against collagen sponge, suggesting that hyaluronic acid sponge has biocompatibility and biodegradation characteristics as well as an appropriate structure to make it suitable as a scaffold for dental pulp regeneration [77] (Figure 4).

 It is also important to clarify neuronal differentiation and neurite outgrowth during regeneration of the dentin-pulp complex. We examined the effects of hyaluronic acid gel on neuronal differentiation of PC12 pheochromocytoma cells, which respond to nerve growth factor (NGF) by extending neurites and exhibit a variety of properties of adrenal medullary chromaffin cells. We applied diluted solutions of 800 kDa hyaluronic acid to NGF-exposed PC12 cells, and noted inhibition of NGF-induced neuronal differentiation of PC12 cells via inhibition of ERK and p38 MAPK activation, caused by the interaction of hyaluronic acid to its receptor, RHAMM [78]. 

 Our results demonstrated that hyaluronic acid sponge is useful for local regeneration of the dentin-pulp complex, whereas hyaluronic acid gel inhibits the differentiation or neurite outgrowth of neurons. *In vivo,* our results showed that hyaluronic acid sponge is gradually biodegraded during the regeneration processes, leaving soluble hyaluronic acid in the regenerated dental pulp. Next, we intend to clarify the biological and physiological behaviors of hyaluronic acid throughout the regeneration the of dentin-pulp complex.

## 4. Future Challenges to Achieve Local Regeneration of the Dentin-Pulp Complex

In our strategy, growth factors and scaffolds are exogenously supplied as bioactive materials, while the source of stem cells that are able to differentiate into odontoblast-like cells and pulp cells is dependent on the residual dental pulp. The vitality of the residual dental pulp is a critical point to achieve local regeneration of the dentin-pulp complex. It is generally accepted that the pulp wound healing proceeds well under conditions of low inflammatory responses by the dental pulp. In addition, regulation of dental pulp infection is another critical point regarding the success of such regeneration therapy. The resin bonding system is commonly used as one of materials showing favorable adhesion to enamel and dentin, and composite resin system with antimicrobial ability was reported [79–81]. These restorative materials may inhibit further bacterial invasion after tissue regeneration of dentin-pulp complex. Furthermore, when we try to induce revascularization and SCAPs and BMMSCs from the apical area into scaffolds at the root canal to regenerate dentin-pulp complex, disinfection of infected root canal systems, as well as proper apical enlargement to permit the induction from periapical tissues, should be successfully established [82]. Local regeneration of the dentin-pulp complex will be accomplished when the regulation mechanisms of pulp inflammation and infection, as well as pulp wound healing and regeneration mechanisms, are clarified.
